# Preparation of Highly Stable DUT-52 Materials and Adsorption of Dichromate Ions in Aqueous Solution

**DOI:** 10.1021/acsomega.2c00373

**Published:** 2022-05-05

**Authors:** Yanqiong Shen, Ruru Duan, Jinjie Qian, Qipeng Li

**Affiliations:** † College of Chemistry and Chemical Engineering, Zhaotong University, Zhaotong 657000, P. R. China; ‡ College of Chemistry and Materials Engineering, Wenzhou University, Wenzhou 325035, P. R. China

## Abstract

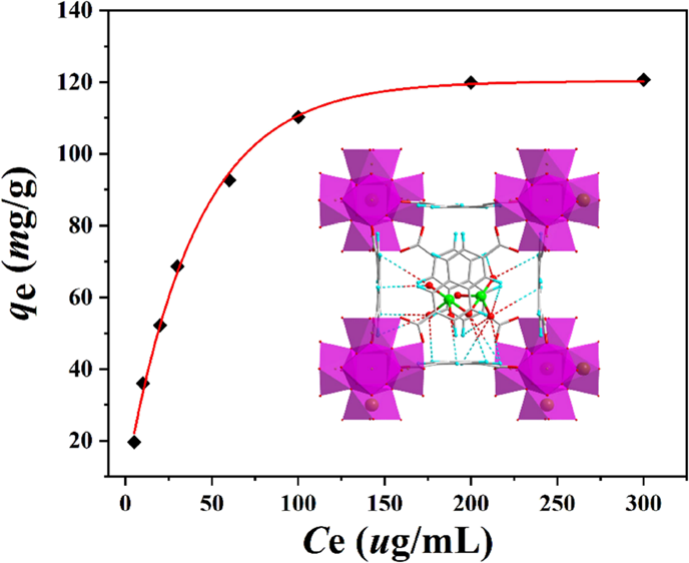

Highly stable **DUT-52** materials were synthesized by the hydrothermal method and well-characterized by X-ray diffraction, thermogravimetric analysis, scanning electron microscopy, and X-ray photoelectron spectroscopy (XPS). In order to systematically study the adsorption of dichromate ions in aqueous solution by the **DUT-52** materials, a single factor experiment, kinetic experiment, thermodynamic experiment, competition ion experiment, and material regeneration experiment were designed. Based on the H-bond interaction between the dichromate ions and the H atoms of a NDC^2–^ ligand, the **DUT-52** materials showed a maximum removal rate of 96.4% and a maximum adsorption capacity of 120.68 mg·g^–1^ with excellent selective adsorption and material regeneration. In addition, the process of adsorption of dichromate ions by the **DUT-52** materials is in accordance with the pseudo second-order kinetics and Langmuir models, and the adsorption mechanism and the important role of the H-bond interaction were reasonably explained using the XPS pattern and theoretical calculation. Accordingly, **DUT-52** can be regarded as a multifunctional material for efficiently removing dichromate ions from the wastewater.

## Introduction

Chromium mainly exists with two valence states (+3 and +6) in nature, and hexavalent chromium ions have strong toxicity and mutational and carcinogenic properties. Hexavalent chromium ions can enter an organism via a variety of ways, lead to damaging of the body, and cause sound hoarse, nasal mucous atrophy, nasal perforation, emphysema and sclerosis diseases, etc.^1−5^ At present, the removal methods of dichromate ions mainly include precipitation, membrane separation, adsorption, ion exchange, biotreatment, and chemical oxidation, as well as a combination of these methods.^6−10^ Some of the above methods endow with severe problems such as high engineering technical difficulty, high potential risk, high cost investment, low adsorption capacity, and weak selection ability. An adsorption method is widely used, due to its easy operation, high efficiency, large adsorption capacity, and recyclable regeneration. However, microporous adsorbent materials (such as activated carbon, large pore resin, natural zeolite, molecular sieve, and silica gel) in the nature have an irregular and relatively complex structure, lacking the functional groups or characteristic structures for capturing dichromate ions in wastewater, and there are no large enough pores and nanocages for capturing and storing dichromate ions.^11−15^

In recent years, a class of metal–organic frameworks (MOFs) were built by inorganic metal ions and organic ligands, compared with the traditional frame structure, molecular sieve, and activated carbon, which show high porosity, large specific surface area, good stability, and simple synthesis process.^16−20^ Structurally speaking, the heavy metal ions enter the nanopore or nanocages of MOF materials and interact with the active sites to realize the function of efficient capture and separation. However, they would have to face various harsh environments requiring high thermal stability of MOF materials and chemical stability in their practical application. To date, only a small number of microporous MOFs have both high thermal and chemical stability, such as zeolitic imidazolate frameworks, material sofistitute Lavoisier frameworks, porphyrin-class MOF and Zr-based MOF materials, and so forth.^16−20^ Recently, highly stable MOF materials have obtained some progress in the capture and isolation of dichromate ions.^21−27^ For example, a cationic porous MOF was prepared by using a neutral triazidazole ligand and AgClO_4_, which is available through anion exchange for high capacity and rapid capture and separation of dichromate ions (Cr_2_O_7_^2–^) in water.^26^ An anionic zirconium-based MOF material (**ZJU-101**) with a specific surface of 561 m^2^·g^–1^, which is much lower than 1862 m^2^·g^–1^ of **MOF-867**, was obtained, but the material can selectively adsorb and separate Cr_2_O_7_^2–^ anions from the aqueous solution by the ion exchange, whose highest adsorption amount is 245 mg·g^-1^.^27^ Although some progress has been achieved in the highly stable MOF materials with regard to the capture and separation of dichromate ions, the design and preparation of highly stable MOFs and their application in the efficient capture and separation of dichromate ions from wastewater remain a challenging work.

In this work, well-known and highly stable **DUT-52** materials were first used for the adsorption of dichromate ions, and the single factor experiment, kinetic experiment, thermodynamic experiment, competition ion experiment, and material regeneration experiment were designed. In addition, the adsorption process of **DUT-52** materials was analyzed, and the dynamic model and thermodynamic model were established, the adsorption process of dichromate ions by **DUT-52** materials was explored, and the adsorption mechanism was reasonably explained by the X-ray photoelectron spectroscopy (XPS) pattern and theoretical calculation. These results provide an idea for the removal and separation of dichromate ions in wastewater.

## Results and Discussion

### Structural and Morphological Characterization

**Figure 1:**
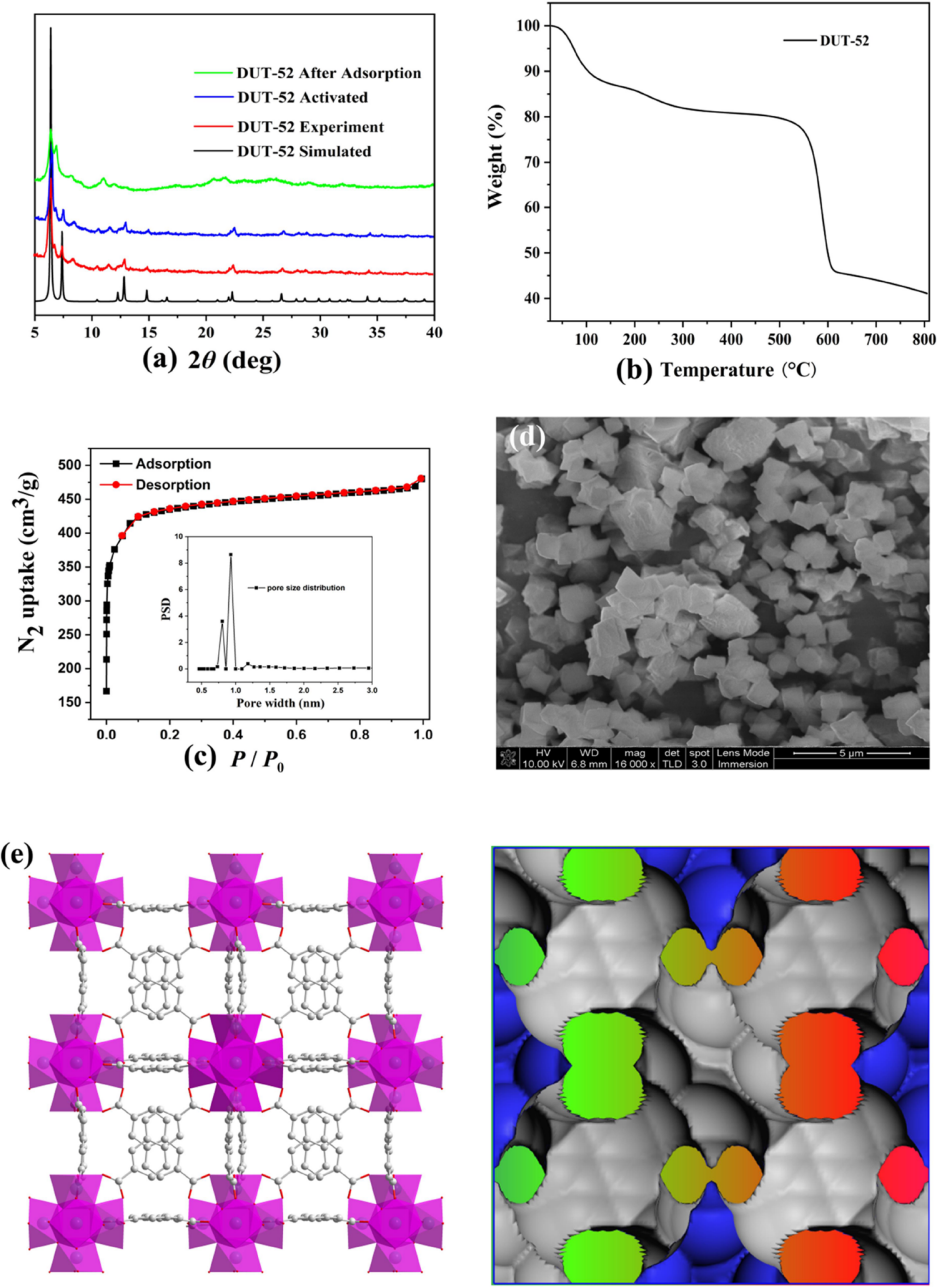
(a) XRD, (b) TGA, (c) N_2_ adsorption and pore size distribution, (d) SEM, and the (e) structure of **DUT-52** materials.

The prepared **DUT-52** materials were characterized by powder X-ray diffraction (XRD), and the characteristic peaks of the experimental and simulated peaks of **DUT-52** materials are basically consistent, which proves the successful formation of porous **DUT-52** materials. The characteristic peak position of the activated sample of **DUT-52** materials was basically unchanged,^28,29^ indicating that the high-temperature activated sample still maintains the crystal state (Figure 1a). **DUT-52** materials were observed as a type of white powder with a regular morphology but uneven size by scanning electron microscopy (SEM) in Figure 1d. The thermal stabilities of the prepared **DUT-52** material samples were characterized by thermogravimetric analysis (TGA). The results showed that the **DUT-52** materials mainly lost the guest solvent molecules in the temperature range of 200–400 °C, while the frameworks began to decompose after 550 °C, indicating that **DUT-52** materials exhibit a high thermal stability (Figure 1b). In addition, **DUT-52** materials can exist stably in 0.1 mol·L^–1^ sodium hydroxide aqueous solution, 0.1 mol·L^–1^ hydrochloric acid solution, H_2_S, H_2_O, and various organic solvents, indicating that **DUT-52** materials also have the exceptional chemical stability.^30−32^ Therefore, **DUT-52** materials have both exceptional chemical stability and high thermal stability.

The structure of **DUT-52** materials is similar to that of UiO series, which is formed by connecting [Zr_6_O_4_(OH)_4_] with 12 NDC^2–^ ligands. This shows that a three-dimensional periodic structure with each octahedral hole shares its triangular window with eight tetrahedral cages.^30−32^**DUT-52** materials have two kinds of channels with the sizes of 14 and 11 Å, respectively (Figure 1c). The Brunauer–Emmett–Teller (BET) value and pore volume of the **DUT-52** materials were characterized with an ASAP2020, and the results show that the N_2_ adsorption and desorption curves of **DUT-52** materials at 77 K conform to Type-I type with a maximum adsorption amount of 480.64 cm^3^·g^–1^, and the calculated BET value is 1685 m^2^·g^–1^, and the pore volume is 0.65 m^3^·g^–1^ (Figure 1d). Compared with the same series of materials, **DUT-52** materials have large specific surface area and pore volume and can be used to adsorb various gas molecules, heavy metal ions, and organic pollutant molecules with the potential application value and application prospects in the environmental field.^30−32^

### Adsorption Experiment

In order to explore the optimal conditions for the adsorption of dichromate aqueous solution by **DUT-52** materials, four single factor optimization experiments were designed, including the dosage of **DUT-52** materials, the initial concentration of dichromate aqueous solution, temperature, and pH values of the dichromate aqueous solutions.

**Figure 2:**
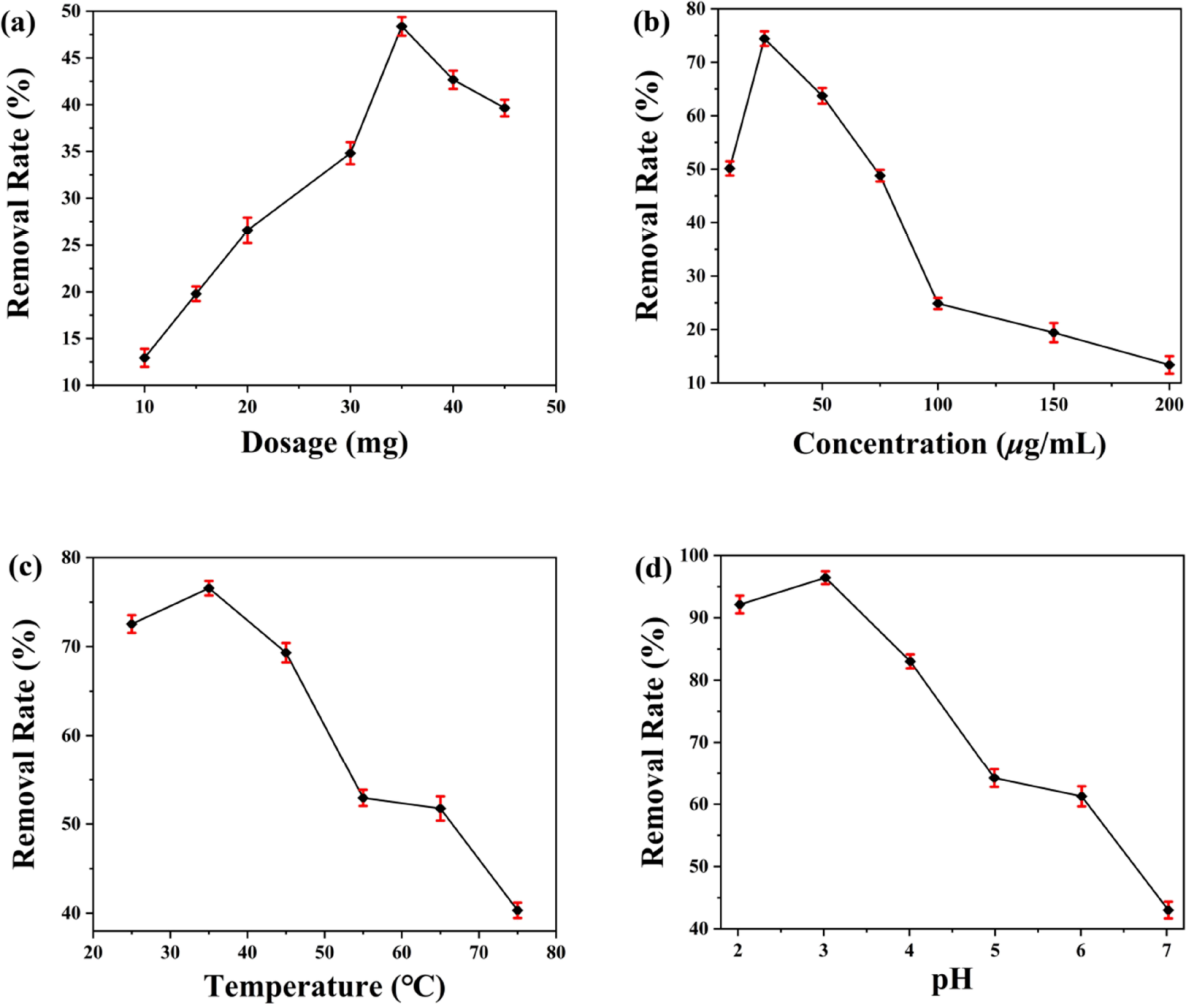
Influence of (a) different dosages, (b) initial concentration of dichromate ions, (c) temperature, and (d) pH on the adsorption by the **DUT-52** materials.

When the dosage of **DUT-52** materials is less than 35 mg, the removal rate of dichromate increases with the dosage of **DUT-52** materials, and when the dosage of **DUT-52** materials is more than 35 mg, the removal rate exhibits a negative correlation (Figure 2a). It is possible that increasing the dosage of **DUT-52** materials can increase the adsorption active sites of the material, while the limited concentration of dichromate will reduce the utilization of the adsorption sites of the material, resulting in a decrease in the removal rate of unit mass adsorbent.^33−40^ Therefore, the optimal dosage of **DUT-5**2 materials is selected as 35 mg with a removal rate of 48.4%. When the initial concentration of dichromate aqueous solution increases from 10 to 25 μg·mL^–1^, the removal rate increased gradually. When the initial concentration of dichromate aqueous solution exceeds 25 μg·mL^–1^, the removal rate decreased gradually with the increase of dichromate aqueous solution concentration (Figure 2b). When the initial concentration of dichromate aqueous solution increases, the removal rate of **DUT-52** materials for dichromate reaches equilibrium, the adsorption amount no longer increases, and the removal rate gradually decreases.^33−41^ Thus, when the concentration of dichromate aqueous solution was 25 μg·mL^–1^, the highest rate of dichromate removal was 74.4%. The optimum adsorption temperature of **DUT-52** materials for dichromate is 35 °C, and the removal rate is 76.6% (Figure 2c). The most suitable adsorption temperature for dichromate adsorption by **DUT-52** materials is 35 °C with a removal rate of 76.6%. When the temperature is in the range of 25–35 °C, the removal rate changes little, while when the temperature is higher than 35 °C, the removal rate decreases gradually with the temperature. Dichromate aqueous solution with a pH value of 3.02 had the highest removal rate of 96.4%. When the pH value is greater than 3.02, the removal rate of dichromate gradually decreased with the increasing solution pH (Figure 2d). When the pH value is between 2 and 5, the dichromate aqueous solution is dominated by HCrO_4_^–^ and Cr_2_O_7_^2–^; when the pH value exceeds 7, the dichromate aqueous solution is dominated by CrO_4_^2–^.^33−41^ Therefore, the removal of the dichromate ions in the aqueous solution is favored for the **DUT-52** materials under acidic conditions.

Therefore, the results show that the optimal adsorption conditions are as follows: the dosage of **DUT-52** materials is 35 mg, the initial concentration is 25 μg·mL^–1^, the adsorption temperature is 35 °C, and the pH value of solution is 3.02, whose removal rate reaches the maximum value of 96.4%.

**Figure 3:**
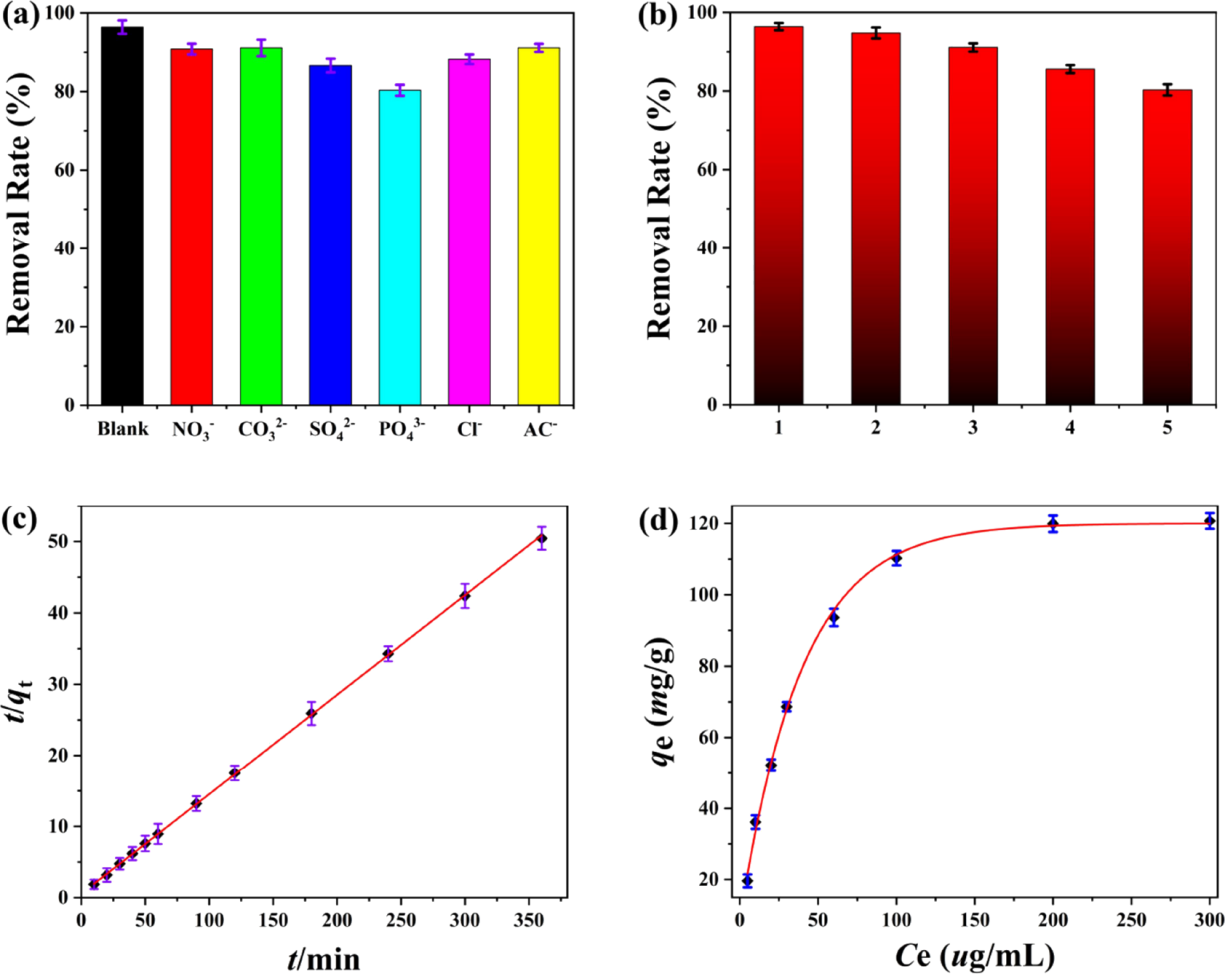
(a) Effect of competing ions on the removal of dichromate by the **DUT-52** materials; (b) cycle experiment of dichromate by the **DUT-52** materials; (c) pseudo second-order dynamic model fitting on the dichromate by the **DUT-52** materials; and (d) adsorption isotherm and Langmuir adsorption mode fitting for the removal of dichromate by the **DUT-52** materials.

Since the industrial wastewater usually contains some co-existing anions such as NO_3_^–^, CO_3_^2–^, SO_4_^2–^, PO_4_^3–^, Cl^–^, AC^–^, and so forth, it is necessary to explore the influence of these co-existing ions on the removal of dichromate ions. The concentrations of the different ions were set to be consistent with the dichromate ions, and the results show that most of the co-existing ions have only mild effects on the adsorption of dichromate ions (Figure 3a), which indicates that **DUT-52** materials can maintain their commendable capture capacity for dichromate ions in the presence of interfering ions. The reusability of **DUT-52** materials after the adsorption of dichromate ions was further investigated, and the methanol–acetic acid was selected as the effluent in the regeneration experiments.^41^ After five recycles, the removal rate of dichromate ions can still reach up to 80.3% and the framework stability of **DUT-52** materials after five recycles still remains intact, which were confirmed by the XRD patterns and SEM (Figure 3b). The experimental results show that the aqueous solution of dichromate with different concentrations gradually reaches equilibrium with the passage of oscillated time, and its adsorption capacity does not increase. When the equilibrium concentration is 300 μg·mL^–1^, its maximum adsorption capacity is 120.68 mg·g^–1^, which has reached the adsorption equilibrium (Figure 3d). Compared with other materials, its maximum adsorption capacity belongs to the moderate level (Table S1).^1,30−41^

The adsorption kinetics can reflect the adsorption rate of dichromate by **DUT-52** materials. According to the data of adsorption time and adsorption capacity, the quasi first-order kinetics and quasi second-order kinetics are fitted by formulas 3 and 4 in which the pseudo first-order kinetic fitting curve is obtained using ln(*q*_e_ – *q*_t_) and *t*, and the pseudo second-order kinetic fitting curve is obtained using *t*/*q*_t_ and *t*, to obtain the kinetic process of adsorption of dichromate by the **DUT-52** materials.^33−41^ The experimental results show that the *R*^2^ value of the pseudo second-order kinetic model is 0.9999 (Figure 3c), indicating that the linear relationship is very good within the performed concentrations.^33−41^ It can be inferred that the adsorption process of dichromate ions by the porous **DUT-52** materials, which conforms to the pseudo second-order kinetic model. The Langmuir and Freundlich models were fitted by eqs 5 and 6 in which *c*_e_ is plotted with the *c*_e_/*q*_e_ to obtain the Langmuir isotherm, while the Freundlich isotherm is obtained by plotting ln*c*_e_ with ln*q*_e_. The experimental results show that the *R*^2^ value of Langmuir isotherm is 0.9987 with a better linearity (Figure 3d). Therefore, the thermodynamic process of dichromate in the **DUT-52** materials is more consistent with the Langmuir model.

Thermodynamic energy can determine whether the reaction can occur, according to eqs 7, 8, and 9, 1/*T* acts as the transverse coordinate and ln*K* acts as the ordinate, to obtain the thermodynamic linear fitting curve. At 308, 318, 328, 338, and 348 K, the Gibbs free energy (Δ*G*) values are all negative (Table S2), which reveals that the adsorption process of **DUT-52** materials on dichromate in aqueous solution is performed spontaneously, and the Δ*G* value gradually increases, indicating that the increased temperature is not conducive to the adsorption process.^33−41^ The enthalpy change (Δ*H*) values in the adsorption process are negative, indicating that the adsorption process is an exothermic process, the entropy change (Δ*S*) values in the adsorption process are positive, indicating that the adsorption process is an entropy increasing process, and its adsorption rate is greater than the desorption rate.^33−41^ Therefore, the process of dichromate adsorption in aqueous solution by **DUT-52** materials is a spontaneous and exothermic process.

### Adsorption Mechanism

**Figure 4:**
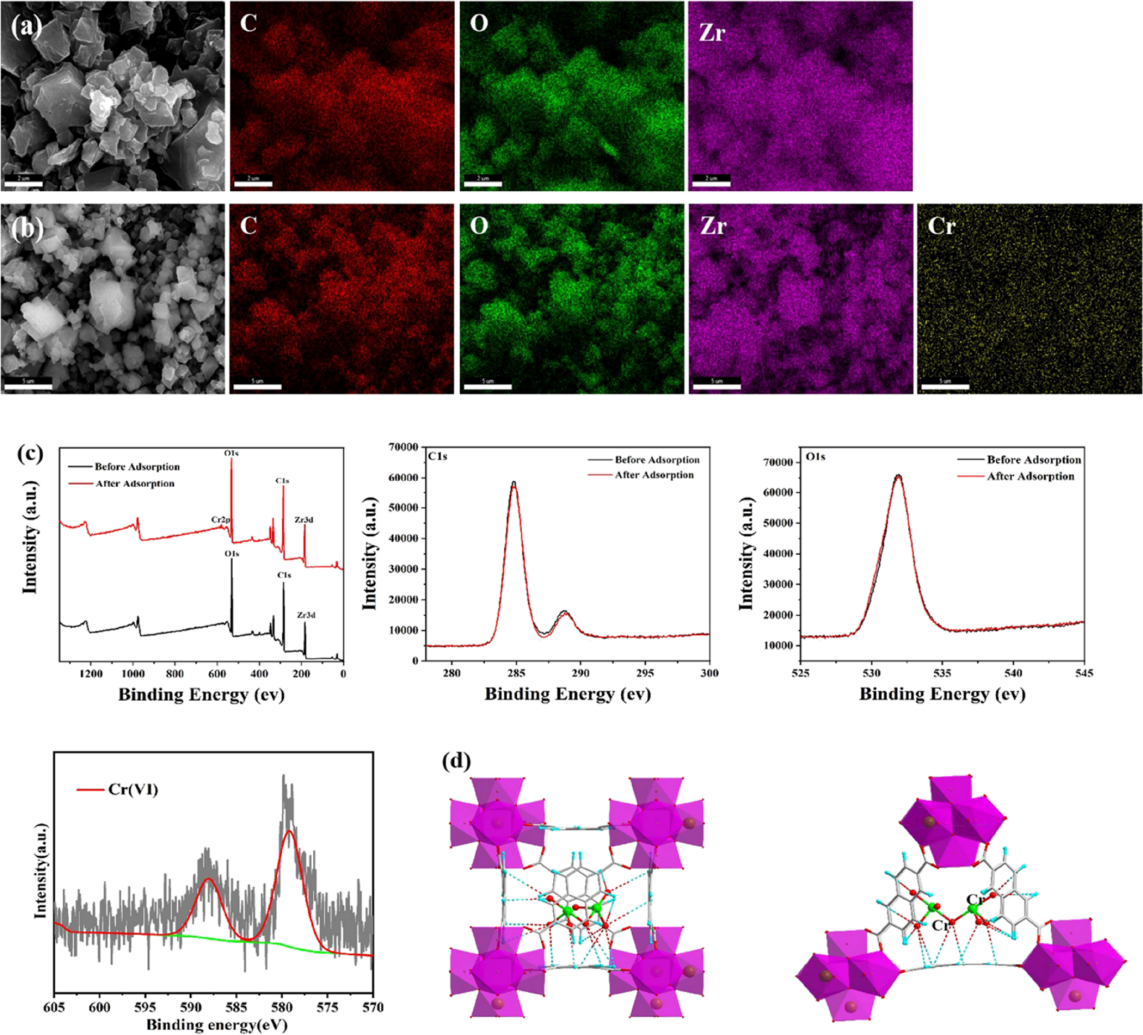
(a) and (b) SEM and elemental mapping of **DUT-52** materials before and after adsorption of dichromate ions; (c) XPS spectra of **DUT-52** materials before and after adsorption of dichromate ions; and (d) adsorption mechanism of dichromate and **DUT-52** with hydrogen bonding interaction.

The sign for the Cr element can be found in the spectrum of elemental mapping and XPS spectra of **DUT-52** materials after the adsorption of dichromate ions (Figure 4a–c), and the peaks at 588.07 and 579.22 eV can be assigned to Cr2p_1/2_ and Cr2p_3/2_, respectively (Figure 4c). From XPS spectra of **DUT-52** materials before and after adsorption of dichromate ions, it can be observed that the peaks of Zr3d and O2s basically had no change, while the peak for C1s has subtle changes, indicating that these H atoms of the NDC^2–^ ligand were involved in the adsorption process. The adsorption locator module of Materials Studios was performed to investigate the adsorption sites of dichromate in the pores of **DUT-52** materials. During the simulation process, the structure of **DUT-52** materials was kept as rigid with atoms frozen at their crystallographic positions. The force field parameters for the framework atoms and adsorbents were both taken from Universal force field, while the partial charges for the framework atoms of **DUT-52** materials were estimated by the QEq method. The possible adsorption mechanism for dichromate ions adsorbed into the pores of **DUT-52** materials mainly involves the H-bond interaction between the *O* atom of dichromate and the *H* atom of the NDC^2–^ ligand (Figure 4d), which plays an important role in the capture of dichromate ions.^1,41^

## Conclusions

The highly stable and porous **DUT-52** materials have been prepared by the hydrothermal method and were characterized by XRD, TGA, SEM, and XPS. The effect of **DUT-52** on the adsorption process of dichromate ions in aqueous solution was explored through the experiments under different conditions. The research results show that **DUT-52** materials show the maximum removal rate of 96.4% and the maximum adsorption capacity of 120.68 mg·g^–1^ with excellent selective adsorption and material regeneration. In addition, the analysis of the kinetic and thermodynamic data shows that the adsorption process of dichromate by **DUT-52** conforms to the pseudo-second-order kinetic model and the Langmuir model, and the adsorption mechanism as well as the important role of H-bond interaction was reasonably explained by the XPS pattern and theoretical calculation. Therefore, **DUT-52** can be regarded as a multifunctional material to efficiently remove dichromate ions from the wastewater, which can provide an idea for the wastewater treatment.

## Materials

### Materials and Methods

All the chemical reagents were commercially purchased and used without further purification. An XRD-6000 X-ray powder diffractor (Shimadzu, Japan); FEI Quanta 200F scanning electron microscope (FEI), thermogravimetric analyzer (Mettel-Toledo, Switzerland); ASAP 2020 (Mike Instruments); PHS-2F pH meter (Shanghai); and UV-2802PCS spectrophotometer (Shanghai Unico Instruments Co., Ltd.) were used; XPS data were collected on an ESCALAB 250 XPS, using Al Kα X-rays as the excitation source.

### Preparation of DUT-52

ZrCl_4_ (1.03 mmol, 230 mg) was dissolved in 20 mL of *N*,*N*′-dimethylformamide (DMF) in a 50 mL polytetrafluoroethylene reactor, which was sonicated for 5 min. Then, 2,6-naphthaleneic acid (216 mg, 1 mmol) was added into the mixture solution and sonicated for 5 min, and then, 3 mL of acetic acid was added, and the mixture was sonicated for 15 min, which was heated in an oven (120 °C) for 24 h and cooled to room temperature.^30−32^ The prepared samples were centrifuged and washed three times with fresh DMF, the solvent was exchanged with ethanol 3 times, the samples were dried and activated under vacuum for 4 h, and the white powder of the activated **DUT-52** materials was obtained.

### Adsorption Experiment

A certain dosage of **DUT-52** materials was added into 10 mL of dichromate aqueous solution with a certain concentration and pH, which were oscillated at different temperatures for 30 min and filtered using a 0.45 μm hydrophobic PTFE membrane, and the residual concentrations of dichromate ions were determined by UV spectrophotometry.
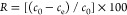
1
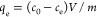
2where *c*_0_ is the initial concentration of the dichromate ions (μg/mL), *c*_e_ is the equilibrium concentration of dichromate ions (μg/mL), *V* is the volume of dichromate ions (mL), *m* is the dosage of adsorbent **DUT-52** materials (mg), and *q*_e_ is the adsorption capacity (μg/mg).

The removal rate (*R*) and the adsorption capacity (*q*_e_) of the **DUT-52** materials were calculated using eqs 1 and 2.

### Regeneration Experiment

The **DUT-52** materials after adsorption of dichromate ions (20 mg) were dispersed into the mixture (40 mL) of methanol and acetic acid (1:1, v/v).^41^ The resulting suspension was stirred for 12 h, and the solid was collected through the concentration. Finally, the collected solid was washed with ethanol and dried and activated under vacuum for 4 h.

### Competition Ion Experiment

First, 35 mg of **DUT-52** materials was added into 10 mL of dichromate aqueous solution (25 μg/mL) with different competition ions (NO_3_^–^, CO_3_^2–^, SO_4_^2–^, PO_4_^3–^, Cl^–^, and AC^–^) and pH (3.02) under 35 °C, which were oscillated for 30 min and filtered using a 0.45 μm hydrophobic PTFE membrane, and then, the residual concentration of dichromate ions was determined.

### Adsorption Kinetics

**DUT-52** materials (35 mg) were added into 10 mL of dichromate aqueous solution with different initial concentrations (10–1000 μg/mL) and pH (3.02) under 35 °C, which were oscillated different times and filtered using a 0.45 μm hydrophobic PTFE membrane, and then, the residual concentration of dichromate ions was determined.
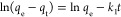
3
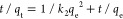
4where *q*_t_ is the adsorption capacity corresponding to *t* (μg/mg), *q*_e_ is the adsorption capacity (μg/mg), *k*_1_ and *k*_2_ are the kinetic rate constants, and *t* is the adsorption time (m).

The kinetic relationship in the adsorption of dichromate aqueous solution by the **DUT-52** materials was calculated based on the quasi-primary kinetic equation (eq 3) and the quasi-secondary kinetic equation (eq 4).

### Adsorption Thermodynamics

First, 35 mg of **DUT-52** materials was added into 10 mL of dichromate aqueous solution with different initial concentrations (10–1000 μg/mL) and pH (3.02) under different temperatures (35–75 °C), which were oscillated for 360 min and filtered using a 0.45 μm hydrophobic PTFE membrane, and then, the residual concentration of dichromate ions was determined.
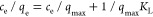
5
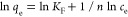
6where *q*_max_ is the maximum saturated adsorption capacity (μg/mg), *q*_e_ is the adsorption capacity (μg/mg), *K*_L_ is the Langmuir adsorption constant related to the adsorption energy, *K*_F_ is the Freundlich adsorption constant related to the adsorption capacity, *n* is a temperature-dependent constant, and *c*_e_ is the equilibrium concentration of dichromate ions (μg/mL).

The thermodynamic relationship in the adsorption of the dichromate aqueous solution by the **DUT-52** materials was calculated based on the Langmuir equation (eq 5) and the Freundlich equation (eq 6).
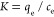
7
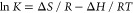
8
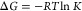
9where *K* is the thermodynamic equilibrium constant, *R* is the gas adsorption constant (8.314 J/mol K), *T* is the absolute temperature (K), and Δ*G*, Δ*S*, and Δ*H* are the Gibbs free energy, entropy, and enthalpy, respectively.

The Δ*G*, Δ*S*, and Δ*H* of the **DUT-52** materials can be calculated according to eqs 7, 8, and 9.

## Supporting Information Available

Cr(VI) ion adsorption performance of some reported MOFs and thermodynamic equilibrium constants (PDF)

## References

[ref1] Li Y. X.; Zhong W. B.; Xie L. H.; Xie Y. B.; Li J. R. Recent Advances in Adsorptive Removal of Cr(VI) Ions by Metal-Organic Frameworks. Chin. J. Inorg. Chem. 2021, 37, 385–400.

[ref2] Wang S. W.; Huang J.; Yang Y.; Hui Y. M.; Ge Y. X.; Larssen T.; Yu G.; Deng S. B.; Wang B.; Harman C. First report of a Chinese PFOS alternative overlooked for 30 years: its toxicity, persistence, and presence in the environment. Environ. Sci. Technol. 2013, 47, 10163–10170. 10.1021/es401525n. 23952109

[ref3] Liu W. Z.; Li J.; Zheng J. Y.; Song Y.; Shi Z. Q.; Lin Z.; Chai L. Y. Different Pathways for Cr(III) Oxidation: Implications for Cr(VI) Reoccurrence in Reduced Chromite Ore Processing Residue. Environ. Sci. Technol. 2020, 54, 11971–11979. 10.1021/acs.est.0c01855. 32905702

[ref4] Gao Q.; Xu J.; Bu X. H. Recent advances about metal-organic frameworks in the removal of pollutants from wastewater. Coord. Chem. Rev. 2019, 378, 17–31. 10.1016/j.ccr.2018.03.015.

[ref5] Wang C. X.; Xia Y. P.; Yao Z. Q.; Xu J. L.; Chang Z.; Bu X. H. Two luminescent coordination polymers as highly selective and sensitive chemosensors for Cr^VI^-anions in aqueous medium. Dalton Trans. 2019, 48, 387–394. 10.1039/C8DT04230F. 30516207

[ref6] Yao Z. Q.; Li G. Y.; Xu J.; Hu T. L.; Bu X. H. A Water-Stable Luminescent Zn^II^ Metal-Organic Framework as Chemosensor for High-Efficiency Detection of Cr^VI^-Anions (Cr_2_O_7_^2–^ and CrO_4_^2–^) in Aqueous Solution. Chem. – Eur. J. 2018, 24, 3192–3198. 10.1002/chem.201705328. 29210125

[ref7] Maurin G.; Serre C.; Cooper A.; Féreyd G. The new age of MOFs and of their porous-related solids. Chem. Soc. Rev. 2017, 46, 3104–3107. 10.1039/C7CS90049J. 28561090

[ref8] Xue Z. Z.; Meng X. D.; Li X. Y.; Han D. S.; Pan J.; Wang G. M. Luminescent Thermochromism and White-Light Emission of a 3D [Ag_4_Br_6_] Cluster-Based Coordination Framework with Both Adamantane-like Node and Linker. Inorg. Chem. 2021, 60, 4375–4379. 10.1021/acs.inorgchem.1c00280. 33729790

[ref9] Bobbitt N. S.; Mendonca M. L.; Howarth A. J.; Islamoglu T.; Hupp J. T.; Farha O. K.; Snurr R. Q. Metal-organic frameworks for the removal of toxic industrial chemicals and chemical warfare agents. Chem. Soc. Rev. 2017, 46, 3357–3385. 10.1039/C7CS00108H. 28345694

[ref10] Zheng T.; Yang Z. X.; Gui D. X.; Liu Z. Y.; Wang X. X.; Dai X.; Liu S. T.; Zhang L. J.; Gao Y.; Chen L. H.; Sheng D. P.; Wang Y. L.; Wu J. D.; Wang J. Q.; Zhou R. H.; Chai Z. F.; Albrecht-Schmitt T. E.; Wang S. A. Overcoming the crystallization and designability issues in the ultrastable zirconium phosphonate framework system. Nat. Commun. 2017, 8, 15369 10.1038/ncomms15369. 28555656 PMC5459948

[ref11] Barea E.; Montoro C.; Navarro J. A. R. Toxic gas removal metal-organic frameworks for the capture and degradation of toxic gases and vapours. Chem. Soc. Rev. 2014, 43, 5419–5430. 10.1039/C3CS60475F. 24705539

[ref12] Zhang Z. H.; Lou Y. F.; Guo C. P.; Jia Q. J.; Song Y. P.; Tian J. Y.; Zhang S.; Wang M. H.; He L. H.; Du M. Metal-organic frameworks (MOFs) based chemosensors/biosensors for analysis of food contaminants. Trends Food Sci. Technol. 2021, 118, 569–588. 10.1016/j.tifs.2021.10.024.

[ref13] Xu P. L.; Cao J. Y.; Yin C.; Wang L. T.; Wu L. Quantum chemical study on the adsorption of megazol drug on the pristine BC3 nanosheet. Supramol. Chem. 2021, 33, 63–69. 10.1080/10610278.2021.1938049.

[ref14] Du X. X.; Tian W. L.; Pan J. H.; Hui B.; Sun J. H.; Zhang K. W.; Xia Y. Z. Piezo-phototronic effect promoted carrier separation in coaxial p-n junctions for self-powered photodetector. Nano Energy 2022, 92, 106694 10.1016/j.nanoen.2021.106694.

[ref15] Wang C. H.; Liu X. L.; Demir N. K.; Chen J. P.; Li K. Applications of water stable metal-organic frameworks. Chem. Soc. Rev. 2016, 45, 5107–5134. 10.1039/C6CS00362A. 27406473

[ref16] Schoenecker P. M.; Carson C. G.; Jasuja H.; Flemming C. J. J.; Walton K. S. Effect of Water Adsorption on Retention of Structure and Surface Area of Metal-Organic Frameworks. Ind. Eng. Chem. Res. 2012, 51, 6513–6519. 10.1021/ie202325p.

[ref17] Ahmadijokani F.; Mohammadkhani R.; Ahmadipouya S.; Rezakazemi M.; Molavi H.; Aminabhavi T. M.; Arjmanda M. Superior chemical stability of UiO-66 metal-organic frameworks (MOFs) for selective dye adsorption. Chem. Eng. J. 2020, 399, 125346 10.1016/j.cej.2020.125346.

[ref18] Jiang H. L.; Feng D. W.; Wang K. C.; Gu Z. Y.; Wei Z. W.; Chen Y. P.; Zhou H. C. An exceptionally stable, porphyrinic Zr metal-organic framework exhibiting pH-dependent fluorescence. J. Am. Chem. Soc. 2013, 135, 13934–13938. 10.1021/ja406844r. 23984878

[ref19] Molavi H.; Hakimian A.; Shojaei A.; Raeiszadeh M. Selective dye adsorption by highly water stable metal-organic framework: Long term stability analysis in aqueous media. Appl. Surf. Sci. 2018, 445, 424–436. 10.1016/j.apsusc.2018.03.189.

[ref20] Ahmadijokani F.; Ahmadipouya S.; Molavi H.; Rezakazemi M.; Aminabhavi T. M.; Arjmand M. Impact of scale, activation solvents, and aged conditions on gas adsorption properties of UiO-66. J. Environ. Manage. 2020, 274, 111155 10.1016/j.jenvman.2020.111155. 32805472

[ref21] Bai Y.; Dou Y. B.; Xie L. H.; Rutledge W.; Li J. R.; Zhou H. C. Zr-based metal-organic frameworks: design, synthesis, structure, and applications. Chem. Soc. Rev. 2016, 45, 2327–2367. 10.1039/C5CS00837A. 26886869

[ref22] Ahmadijokani F.; Tajahmadi S.; Bahi A.; Molavi H.; Rezakazemi M.; Ko F.; Aminabhavi T. M.; Arjmand M. Ethylenediamine-functionalized Zr-based MOF for efficient removal of heavy metal ions from water. Chemosphere 2021, 264, 128466 10.1016/j.chemosphere.2020.128466. 33065327

[ref23] Peng Y. G.; Huang H. L.; Zhang Y. X.; Kang C. F.; Chen S. M.; Song L.; Liu D. H.; Zhong C. L. A versatile MOF-based trap for heavy metal ion capture and dispersion. Nat. Commun. 2018, 9, 187 10.1038/s41467-017-02600-2. 29335517 PMC5768720

[ref24] Wu S. B.; Ge Y. J.; Wang Y. Q.; Chen X.; Li F. F.; Xuan H.; Li X. Adsorption of Cr(VI) on nano UIO-66-NH_2_ MOFs in water. Environ. Technol. 2018, 39, 1937–1948. 10.1080/09593330.2017.1344732. 28625105

[ref25] Fu H. R.; Xu Z. X.; Zhang J. Water-stable Metal-Organic Frameworks for fast and high dichromate trapping *via* single-crystal-to-single-crystal ion exchange. Chem. Mater. 2015, 27, 205–210. 10.1021/cm503767r.

[ref26] Li X. X.; Xu H. Y.; Kong F. Z.; Wang R. H. A cationic Metal-Organic Framework consisting of nanoscale cages: capture, separation and luminescent probing of Cr_2_O_7_^2–^ through a single-crystal to single-crystal process. Angew. Chem., Int. Ed. 2013, 52, 13769–13773. 10.1002/anie.201307650. 24174403

[ref27] Zhang Q.; Yu J. C.; Cai J. F.; Zhang L.; Cui Y. J.; Yang Y.; Chen B. L.; Qian G. D. A porous Zr-cluster-based cationic metal-organic framework for highly efficient Cr_2_O_7_^2–^ removal from water. Chem. Commun. 2015, 51, 14732–14734. 10.1039/C5CC05927E. 26291500

[ref28] Chen C. X.; Yang S. S.; Ding J.; Wang G. Y.; Zhong L.; Zhao S. Y.; Zang Y. N.; Jiang J. Q.; Ding L.; Zhao Y.; Liu L. M.; Ren N. Q. Non-covalent self-assembly synthesis of AQ_2_S@rGO nanocomposite for the degradation of sulfadiazine under solar irradiation: The indispensable effect of chloride. Appl. Catal., B 2021, 298, 120495 10.1016/j.apcatb.2021.120495.

[ref29] Li G. B.; Huang S. Q.; Zhu N. W.; Yuan H. P.; Ge D. D. Near-infrared responsive upconversion glass-ceramic@BiOBr heterojunction for enhanced photodegradation performances of norfloxacin. J. Hazard. Mater. 2021, 403, 123981 10.1016/j.jhazmat.2020.123981. 33265020

[ref30] Zhang W. J.; Huang H. L.; Liu D. H.; Yang Q. Y.; Xiao Y. L.; Ma Q. T.; Zhong C. L. A new metal-organic framework with high stability based on zirconium for sensing small molecules. Microporous Mesoporous Mater. 2013, 171, 118–124. 10.1016/j.micromeso.2013.01.003.

[ref31] Bon V.; Senkovska I.; Weiss M. S.; Kaskel S. Tailoring of network dimensionality and porosity adjustment in Zr-and Hf-based MOFs. CrystEngComm 2013, 15, 9572–9577. 10.1039/c3ce41121d.

[ref32] Huang H. L.; Zhang W. J.; Yang F.; Wang B.; Yang Q. Y.; Xie Y. B.; Zhong C. L.; Li J. R. Enhancing CO_2_ adsorption and separation ability of Zr(IV)-based metal-organic frameworks through ligand functionalization under the guidance of the quantitative structure-property relationship model. Chem. Eng. J. 2016, 289, 247–253. 10.1016/j.cej.2015.12.100.

[ref33] Li L. L.; Feng X. Q.; Han R. P.; Zang S. Q.; Yang G. Cr(VI) removal via anion exchange on a silver-triazolate MOFs. J. Hazard. Mater. 2017, 321, 622–628. 10.1016/j.jhazmat.2016.09.029. 27694026

[ref34] Soltani R.; Marjani A.; Shirazian S. A hierarchical LDH/MOF nanocomposite: single, simultaneous and consecutive adsorption of a reactive dye and Cr(VI). Dalton Trans. 2020, 49, 5323–5335. 10.1039/D0DT00680G. 32248208

[ref35] Tripathy S. P.; Subudhi S.; Das S.; Ghosh M. K.; Das M.; Acharya R.; Parida K. Hydrolytically stable citrate capped Fe_3_O_4_@UiO-66-NH_2_ MOF: A hetero-structure composite with enhanced activity towards Cr(VI) adsorption and photocatalytic H_2_ evolution. J. Colloid Interface Sci. 2022, 606, 353–366. 10.1016/j.jcis.2021.08.031. 34392031

[ref36] Wang X.; Liu W.; Fu H. F.; Yi X. H.; Wang P.; Zhao C.; Wang C. C.; Zheng W. W. Simultaneous Cr(VI) reduction and Cr(III) removal of bifunctional MOF/Titanate nanotube composites. Environ. Pollut. 2019, 249, 502–511. 10.1016/j.envpol.2019.03.096. 30928522

[ref37] Luo M. B.; Xiong Y. Y.; Wu H. Q.; Feng X. F.; Li J. Q.; Luo F. The MOF Technique: A Significant Synergic Effect Enables High Performance Chromate Removal. Angew. Chem., Int. Ed. 2017, 56, 16376–16379. 10.1002/anie.201709197. 29094516

[ref38] Yang P. F.; Shu Y. F.; Zhuang Q. X.; Li Y. S.; Gu J. L. Metal-Organic Frameworks Bearing Dense Alkyl Thiol for the Efficient Degradation and Concomitant Removal of Toxic Cr(VI). Langmuir 2019, 35, 16226–16233. 10.1021/acs.langmuir.9b03057. 31702161

[ref39] Dai Q. G.; Shen K.; Deng W.; Cai Y. P.; Yan J. R.; Wu J. Y.; Guo L. M.; Liu R.; Wang X. Y.; Zhan W. C. HCl-Tolerant HxPO_4_/RuOx-CeO_2_ Catalysts for Extremely Efficient Catalytic Elimination of Chlorinated VOCs. Environ. Sci. Technol. 2021, 55, 4007–4016. 10.1021/acs.est.0c08256. 33666414

[ref40] Shi C. W.; Wu Z.; Yang F.; Tang Y. Janus particles with pH switchable properties for high-efficiency adsorption of PPCPs in water. Solid State Sci. 2021, 119, 106702 10.1016/j.solidstatesciences.2021.106702.

[ref41] Zheng M. Q.; Zhao X. D.; Wang K. K.; She Y. B.; Gao Z. Q. Highly Efficient Removal of Cr(VI) on a Stable Metal-Organic Framework Based on Enhanced H-Bond Interaction. Ind. Eng. Chem. Res. 2019, 58, 23330–23337. 10.1021/acs.iecr.9b04598.

