# Supplementary material for: Preparation of Highly Stable DUT-52 Materials and Adsorption of Dichromate Ions in Aqueous Solution
Source: ACS Omega. 2022 May 5;7(19):16414–21. doi: 10.1021/acsomega.2c00373 (PMC9118407; doi:10.1021/acsomega.2c00373)
Supplement: Supplementary file 1 — ao2c00373_si_001.pdf [file ao2c00373_si_001.pdf]

Supporting Information

Preparation of Highly stable DUT-52 Materials and Adsorption of Dichromate ions in Aqueous Solution

Yanqiong Shen[†], *Ruru Duan*[†], *Jinjie Qian*[‡]* *and Qipeng Li*[†]*

[†] *College of Chemistry and Chemical Engineering, Zhaotong University, Zhaotong, 657000, P. R. China*

[‡] *College of Chemistry and Materials Engineering, Wenzhou University, Wenzhou, 325035, P. R. China*

***Correspondence Author:** *jinjieqian@wzu.edu.cn* and *qpli@ztu.edu.cn*

Table S1 Cr(VI) ions adsorption performance of some reported MOFs

MOFs	Adsorbate	Adsorption quantity / (mg/g)	Ref
ABT-$(ClO_4)_2$	$Cr_2O_7^{2-}$	271	1-5
FIR-53	$Cr_2O_7^{2-}$	74.2	1-5
FIR-54	$Cr_2O_7^{2-}$	103	1-5
Cu-BTC	CrO_4^{2-}	48	1-5
$[Cu_4O(BDC)]_n$	Cr(VI)	43.9	1-5
ZJU-101	$Cr_2O_7^{2-}$	245	1-5
MONT-1	$Cr_2O_7^{2-}$	211.8	1-5
TMU-30	$HCrO_4^-$	145	1-5
NU-1000	$Cr_2O_7^{2-}$	76.8	1-5
UIO-66-NH_2	Cr(VI)	32.36	1-5
MIL-100(Fe)-Na_2CO_3	$HCrO_4^-$	46.02	1-5
ZIF-8@CA	Cr(VI)	41.8	1-5
BUT-39	$Cr_2O_7^{2-}$	215	1-5
TMU-66	$HCrO_4^-$	60.24	1-5
MOF-801	$HCrO_4^-$	156.2	1-5
Cu^{II}-MOF	$HCrO_4^-$	190	1-5
SCNU-Z1-C1	CrO_4^{2-}/$Cr_2O_7^{2-}$	149.5/305.8	1-5
Zr-MSA	$HCrO_4^-$	202.0	1-5
Zr-DMSA	$HCrO_4^-$	138.7	1-5
NiCo-LDH	Cr(VI)	99.9	1-5
UPC-50	$Cr_2O_7^{2-}$	56.8	1-5
UIO-66	$Cr_2O_7^{2-}$	60.2	1-5
Dy-MOF	$Cr_2O_7^{2-}$	62.88	1-5
JLU-MOF60	$Cr_2O_7^{2-}$	149	1-5
**DUT-52**	$\mathbf{Cr_2O_7^{2-}}$	**120.68**	**This work**

Table S2 Thermodynamic equilibrium constant

Concentration (ug/mL)	R^2	△*H* (kJ/mol)	△*S* $J\cdot mol^{-1}\cdot K^{-1}$	△*G* (kJ/mol)				
				308 K	318 K	328 K	338 K	348 K
25	0.9992	-48.1638	132.3857	-7.1953	-5.4023	-4.5109	-4.0871	-1.9534
50	0.9978	-46.4021	152.0206	-6.5230	-5.8843	-5.9459	-5.0845	-3.3532
75	0.9971	-29.5853	82.1594	-3.0438	-3.1156	-2.1395	-2.1784	-1.8337
100	0.9925	-26.4983	74.8839	-3.0438	-2.3751	-1.4040	-1.1128	-0.7351

Reference

(1) Zhang, W. J.; Huang, H. L.; Liu, D. H.; Yang, Q. Y.; Xiao, Y. L, Ma, Q. T.; Zhong, C. L. A new metal-organic framework with high stability based on zirconium for sensing small molecules. *Micropor. Mesopor. Mat.* **2013**, 171, 18124.

(2) Bon, V.; Senkovska, I.; Weiss, M. S.; Kaskel, S. Tailoring of network dimensionality and porosity adjustment in Zr-and Hf-based MOFs. *CrystEngComm.* **2013**, 15(45), 9572-9577.

(3) Huang, H. L.; Zhang, W. J.; Yang, F.; Wang, B.; Yang, Q. Y.; Xie, Y. B.; Zhong, C. L.; Li, J. R. Enhancing CO_2 adsorption and separation ability of Zr(IV)-based metal-organic frameworks through ligand functionalization under the guidance of the quantitative structure-property relationship model. *Chem. Eng. J.* **2016**, 289, 247-253.

(4) Li, Y. X.; Zhong, W. B.; Xie, L. H.; Xie, Y. B.; Li, J. R. Recent Advances in Adsorptive Removal of Cr(VI) Ions by Metal-Organic Frameworks. *Chinese J. Inorg. Chem.* **2021**, 37(3), 385-400.

(5) Zheng, M. Q.; Zhao, X. D.; Wang, K. K.; She, Y. B.; Gao, Z. Q. Highly Efficient Removal of Cr(VI) on a Stable Metal-Organic Framework Based on Enhanced H-Bond Interaction. *Ind. Eng. Chem. Res*, **2019**, 58, 23330-23337.
